# Purification of Tannin

**Published:** 1869-08

**Authors:** 


PURIFICATION OF TANNIN.—M. Heintz (*Zeitschrift fur Chemie*), purifies commercial tannic acid by dissolving it in water, and agitating it rapidly with ether that has been well purified. The decanted solution is then filtered, the ether evaporated, and the tannin obtained free from odor or evaporation.

